# Oral fluid supplementation for the prevention of post-dural puncture headache: A noninferiority randomized controlled trial

**DOI:** 10.1371/journal.pone.0319481

**Published:** 2025-03-12

**Authors:** Emmanuelle Cartron, Christelle Volteau, Maxime Leroy, Annastasia Voisine, Jérôme Emmanuel Dauvergne, Céline Ballet, Jean-Philippe Talarmin, Marie-Annick Quéau, Marylène Catinault, Emmanuelle Gazeau, Carole Haubertin, Romain Charreau, David Boutoille

**Affiliations:** 1 Department of Nursing, Université Paris Cité, Paris, France; 2 Inserm - UMR 1123, ECEVE, F-75010 Paris, France; 3 Direction de la Recherche et de l’Innovation, CHU Nantes, Nantes, France; 4 Biostatistics Department, CHU Lille, Lille France; 5 Service d’anesthésie réanimation, Hôpital Laënnec, CHU Nantes, Nantes, France; 6 Service des urgences, CHD Vendée, La Roche Sur Yon, France; 7 Service des Maladies Infectieuses, CH Cornouailles, Quimper, France; 8 CH Le Mans,1 Le Mans, France; 9 Service de médecine - polyneurologie, Cholet, France; 10 Emergency Department, CHU Angers, Angers, France; 11 Service de neurologie, CHR Orléans, Orléans, France; 12 Department of Infectious Diseases, CHU Nantes, Nantes, France; Mayo Clinic, UNITED STATES OF AMERICA

## Abstract

**Aim(s):**

To investigate the impact of the absence of specific advice for oral fluid intake, compared to supplementation water intake on the occurrence of post-dural puncture headache.

**Design:**

A prospective, open-label, non-inferiority, multicenter trial including hospitalized patients requiring a diagnostic lumbar puncture in seven hospitals in France.

**Methods:**

Patients were randomly allocated (1:1) either to receive no specific advice on oral fluid intake (FREE-FLUID), or to be encouraged to drink 2 liters of water (CONTROL) within the 2 hours after lumbar puncture. The primary outcome was the post-dural puncture headache rate within the 5 days after lumbar puncture, with a non-inferiority margin of 10%. The secondary outcome was the time-to-post-dural puncture headache onset between Day 0 and Day 5.

**Results:**

From November 2016 and July 2019, we have included 554 participants. The primary outcomes occurs in 33.1% patients in the FREE-FLUID group, versus 38.0% in the CONTROL group with adjusted difference of 3.7%.

**Conclusion:**

Among patients who had lumbar puncture, our study shows the noninferiority of the absence of specific advice on water intake after a lumbar puncture, compared with advice to increase oral fluid to prevent a post-dural puncture headache.

**Impact:**

The value of questioning the appropriateness of non-evidence-based nursing care may allow time to be devoted to more relational and comforting care.

**Reporting method:**

The study adheres to the CONSORT reporting guidelines.

**Patient or public contribution:**

No patient or public contribution.

**Trial Registration:**

Clinical Trials.gov (NCT02859233, August 9, 2016).

## Introduction

The post-dural puncture headache is a frequent complication of lumbar puncture, defined as any orthostatic headache occurring within 5 days after a lumbar puncture. It may be associated with neck stiffness and/or subjective hearing symptoms [1]. It mostly appears (90%) within 72 hours after the lumbar puncture, and even 66% within 48 hours [2,3]. In 95% of cases, the headache disappears spontaneously within a week [4].

Because bed rest is an analgesic position, post-dural puncture headache can increase the length of hospital stay, and can be responsible for work stoppages [5]. Occurrence of post-dural puncture headache can reach 36%, according to the presence of some patient-related risk factors, such as being aged between 18 and 30 years, being a woman, and having a history of headaches [6]. Moreover, post-dural puncture headache is rare over the age of 60 [3]. Other risk factors are operator-related, such as the gauge of the needle, the bevel point needle, its orientation, and the experience of the operator [6,7]. Although atraumatic needles are not routinely used [8], they remain one of the most effective preventive measures [9]. Whereas some providers suggest a bed rest to prevent post-dural puncture headache, this measure has already been assessed by a large number of research studies, which concluded a lack of benefit of this procedure after lumbar puncture to prevent the post-dural puncture headache [5].

### Background

Oral fluid therapy is commonly used in clinical practice to prevent the post-dural puncture headache [10–14]. Additional fluid intake was suggested to compensate the decrease of intracranial pressure due to the cerebrospinal fluid loss. However, that approach is not supported by robust data [5]. An old single-center study assessed the benefit of increasing oral fluid intake after a lumbar puncture, with a randomized controlled study of 100 patients [15]. It compared daily fluid intakes of 1,5 liters versus 3 liters the following days after lumbar puncture. The results suggested that there were no differences between the two strategies in the occurrence of post-dural puncture headache. Moreover, several risks of bias hampered this study from initiating a real change in professional practice [5]. To date, the benefit of supplementary fluids to prevent post-dural puncture headache is not an evidence based practice. In addition, beyond the discomfort generated for the patient, this strategy adds an extra burden on nurses as it requires informing the patient, providing the extra drinks, and ensuring that they comply with recommendations.

### Aim and objectives

To investigate the impact of not advising a supplementation of oral intake versus encouraging 2 liters of fluid intakes on the occurrence of post-dural puncture headache. The primary objective was to assess the noninferiority of no fluid intake advice in comparison to a supplementation of oral fluid with 2 liters of water within 2 hours after lumbar puncture. The study hypothesis was that no advice on water intake (FREE FLUID group) was non inferior to the practice of supplementation oral fluid, with a non-inferiority margin of 10% [16].

## Materials and methods

### Ethics statement

This study was conducted in accordance with the French West Ethic Committee (ref. 20/16), the French Agency for drug safety (2016 A00560-51), and with Good Clinical Research Practices. All persons voluntarily participated in this study between the 11/08/2016 and the 07/23/2019, after receiving a written information note. Informed written consent was gained from each participant.

### Study design

This multicenter, randomized controlled open label clinical trial was reported in accordance with the Extension of the Consolidated Standards of Reporting Trials (CONSORT) 2010 for reporting of Noninferiority and Equivalence Randomized Trials guidelines [16,17]. Patients were randomly allocated to either the FREE FLUID or CONTROL group with 1:1 ratio, using the Ennov Clinical® Software (Paris, France). The randomization was stratified by center. A randomization list was generated with fixed blocks of size 6.

### Outcomes and definitions

The primary outcome was the occurrence of post-dural puncture headache according to the Headache Classification Committee of the International Headache Society, defined by orthostatic headache within the 5 days after a successful dural puncture, accompanied by neck stiffness and/or subjective hearing symptoms. The secondary outcome was the time-to- post-dural puncture headache onset between D0 and D5.

### Study setting and sampling.

The study was conducted in seven hospitals in France: two teaching hospitals and five non-teaching hospitals (S1 Table). The rate of post-dural puncture headache is assumed to be 20% in both groups, according to studies on diagnostic lumbar puncture [4] and to an internal survey. In the lack of a previous study comparing supplementation of oral intake with placebo, the noninferiority margin was set at 10% [17], the alpha (one-sided) and beta risks are set at 2.5% and 20% respectively, and a maximum attrition rate of 10% is expected. With these assumptions, 277 patients are required in each group, thus a total of 554 patients.

### Inclusion and/or exclusion criteria.

Hospitalized patients aged 18 to 60 and requiring a diagnostic lumbar puncture for acute or chronic medical conditions were included. Patients with a contraindication for increased oral fluid intake, a previous lumbar puncture within the 5 days prior to study enrollment, parenteral fluid intake greater than 266 ml within the 2 hours (i.e., 1 liter per day) after the lumbar puncture, or enteral artificial feeding were not eligible for inclusion. Pregnancy, social protected measures, and lack of health insurance were also non-inclusion criteria.

### Intervention

The lumbar puncture was performed according to the usual clinical practices of each operator. All characteristics of lumbar puncture procedure were recorded. To ensure homogenous practices in all participating centers, information were provided to investigators that detailed how to formulate post- lumbar puncture recommendations (S2 Table).

After the lumbar puncture, according to the randomization group, the patient was either provided with 4 water bottles of 0,5 liters and recommendation to drink them within the 2 hours following the lumbar puncture (CONTROL group), or one water bottle of 0,5 liters with no specific advice on fluid intake (FREE-FLUID group). Patients in the CONTROL group, received a notebook to record their intake at 1-hour, and 2-hour intervals after the lumbar puncture, help them to remind to drink. Patients in the FREE-FLUID group were notified that the water bottle was provided to quantify fluid intake as part of routine monitoring procedures after post-dural puncture headache, and the healthcare team had to refrain their usual advice on water intake. All other usual procedures of the center regarding patient monitoring were maintained according to the standards of care. After 2 hours, all patients were asked to complete a headache diary until D5. In the case of hospital discharge before D5, a telephone interview was programmed. If the patient declared that they still had headaches at the time of the telephone call, another interview was scheduled after day 8 (D8) to advise them to consult a doctor.

### Data collection

Demographic data, reasons for lumbar puncture, and descriptive data regarding clinical status of the patients were collected from the patient file according to the institution’s standard procedures. Data concerning the lumbar puncture included the needle characteristics (type and size), puncture characteristics (needle insertion and withdrawal modalities, the number of puncture attempts), and the volume of cerebrospinal fluid drawn were collected from the doctor who performed the lumbar puncture. Intervention data as well as the volume of oral fluid intake within the 2 hours after the lumbar puncture was also recorded. The post-intervention data were the occurrence of post-dural puncture headache, its occurrence day, and duration, as reported by the patient during the telephone interviews. Participants were included between the 08 November 2016, and the 23 July 2019. The end of the follow up was on the 28 August 2019.

### Data analysis

As recommended for non-inferiority clinical trial, study analyses were performed on both the “Intent to Treat” (ITT) and “Per Protocol” (PP) population [16,17]. The ITT population was defined as all subjects randomized in the study regardless of the treatment compliance, treatment received or breaches of the protocol. The PP population was defined as randomized patients excluding those who had at least one major protocol deviation (no lumbar puncture carried out or failed, missing data on the presence of post-dural puncture headache, volume of intake less than 0.5 liters in the CONTROL group. Missing data were imputed as absence of post-dural puncture headache. Three analysis of sensitivity were conducted: i) Analysis on PP population, ii) Analysis on ITT population with multiple imputation of the missing data and iii) Analysis on ITT population with simple imputation (as primary analysis) and adjustments of baseline risk factors. For multiple imputation, stratification factor, group of randomization, baseline factors associated with primary endpoint, baseline factors associated with groups, and baseline factors associated with missing status for primary endpoint were considered. Baseline risk factors were: sex, age, type of needle used for lumbar puncture (pencil point repositioned stylet, pencil point without repositioning of the stylet, bevel point with tangential retraction, bevel point without tangential retraction), needle diameter and volume of cerebrospinal fluid collected (continuous or in classes: < 17, 17–30,> 30mL) [6].

Primary analysis: the proportion of post-dural puncture headache was estimated in the two groups. A two-sided 95% confidence interval (CI) for the difference between post-dural puncture headache in the two groups (FREE-FLUID – CONTROL) was calculated with a generalized linear mixed model (logistic regression) with stratification factor on center included as random effect. The upper limits of the CIs were to be compared to the non-inferiority margin set at 10%.

Secondary analyses: linear mixed model was used to compare the time-to- post-dural puncture headache onset between the two groups, with stratification factor on center included as random factor. Duration of the post-dural puncture headache was also compared.

Continuous data were presented as mean and its standard deviation, or median and interquartile range in case of asymmetric distribution. Categorical data were presented by number (%). Statistical analysis has been performed with SAS® software version 9.4.

## Results

### Characteristics of the sample

**Fig 1:**
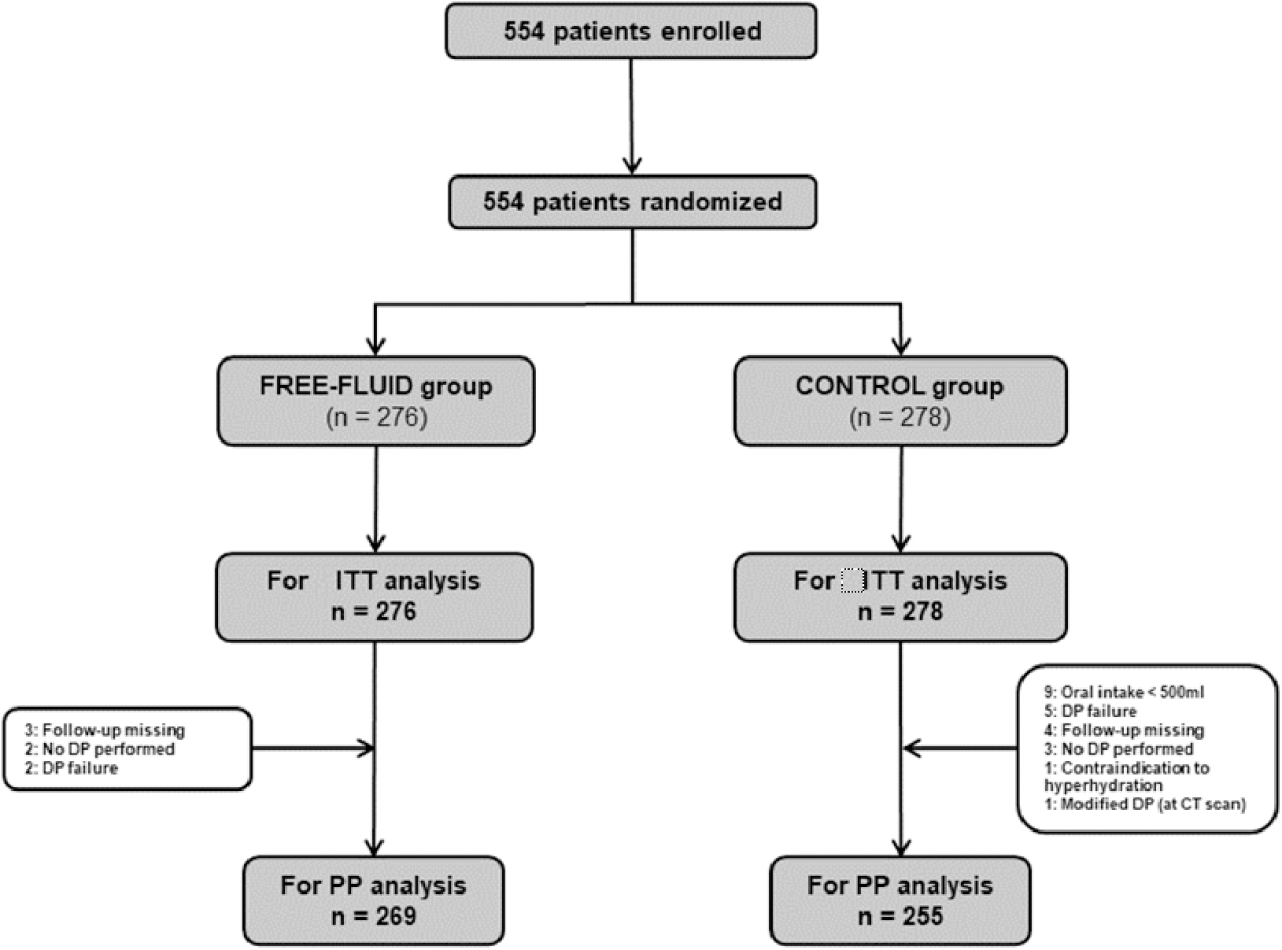
Flowchart Diagram of Participants in the study. Legend: Boxplots indicate the median value (thick line inside the box), and the first and third quartile (thick line around the box). The circles represent outliers’ data. LP: lumbar puncture.

Between November 2016 and July 2019, 554 patients were enrolled in the study, and randomized (276 in the FREE-FLUID group, and 278 to the CONTROL group). Of the 554 patients in the modified intent-to-trait population, 30 patients (7 in the FREE FLUID group, and 23 in the CONTROL group) were not included in the PP population (Fig 1). Baseline patient and lumbar puncture characteristics are presented in Table 1. Five patients did not have any lumbar puncture. Among included patients, 372 (67.1%) were discharged the day after the lumbar puncture, and 55 (9.9%) were still hospitalized at day 5.

**Table 1:** Baseline characteristics of patients.

Characteristics	FREE-FLUID group N = 276	CONTROL group N = 278
Age, median [IQR]	40 [23;49]	43 [32;50]
Female sex, n (%)	170 (61.6%)	179 (64.4%)
BMI, median [IQR]	23.9 [21.4;27.8]	23.7 [21.3;27.7]
Indications of the lumbar puncture, n (%)		
Infectious disease	66 (23.9%)	69 (25.0%)
Inflammatory of nervous system	167 (60.5%)	171 (62.0%)
Tumoral processus	5 (1.8%)	0
Intracranial hemorrhage	33 (12.0%)	31 (11.2%)
Intracranial hypertension	2(1.5%)	3 (1.1%)
*Missing*	*(n = 1)*	*(n = 4)*
Needle design type		
Cutting tip	150 (55.4%)	166 (61.7%)
Pencil point tip (atraumatic needle)	121 (44.6%)	103 (38.3%)
*Missing*	*(n = 5)*	*(n = 9)*
Needle gauge		
Gauge 20	13 (4.9%)	8 (3.0%)
Gauge 21	20 (7.5%)	13 (4.8%)
Gauge 22	124 (46.3%)	148 (54.6%)
Gauge 24	8 (3%)	7 (2.6%)
Gauge 25	101 (37.7%)	93 (34.3%)
Gauge 26	1 (0.4%)	0 (0%)
Gauge 27	1 (0.4%)	2 (0.7%)
* Missing*	*(n = 8)*	*n(=7)*
Needle insert withdrawal		
Not tangential to the dural fibers	44 (29.5%)	42 (26.2%)
Tangential to the dural fibers	105 (70.5%)	118 (73.8%)
Stylet reintroduction	177 (66.3%)	175 (65.5%)
* Missing*	*(n = 127)*	*(n = 118)*
Number of puncture attempts, median [IQR]	1 [1;2]	1 [1;2]
CSF (number of drops), median [IQR]	75 [60;100]	80 [58;120]

Legend: Data are presented as numbers (%), median (interquartile range). BMI: body mass index, LP: lumbar puncture, CSF: cerebrospinal fluid, IQR: interquartile range.

Diagnosis suspected inflammatory disorders of the central nervous system (multiple sclerosis, retrobulbar ocular neuritis, or neurological deficit without diagnostic hypothesis) was the most frequent reason of lumbar puncture.

### Intervention: hyperhydration, or free hydration

**Fig 2:**
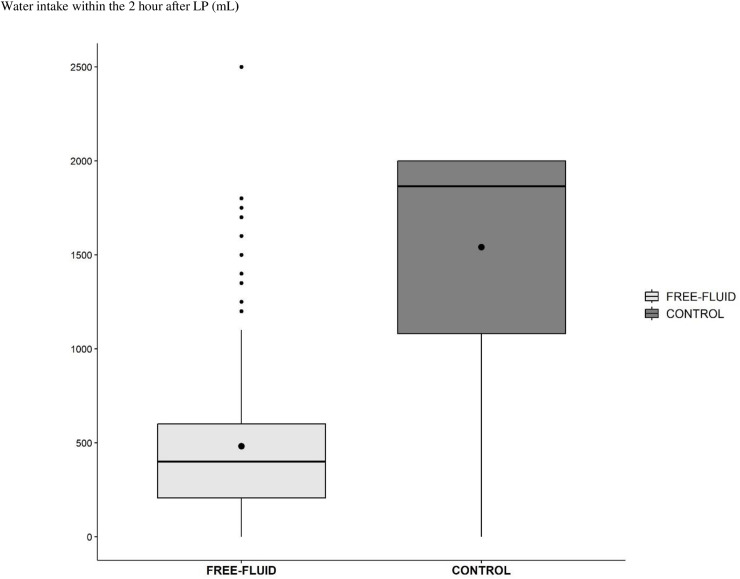
Volume of oral fluid intake within the 2 hours after LUMBAR PUNCTURE with respect of randomization group.

The median oral fluid intake within the first 2 hours following the lumbar puncture was 0.4 liters [IQR, 0,205; 0,600] versus 1.875 liters [IQR, 1,075; 2,000] in the FREE-FLUID group and CONTROL group respectively (Fig 2). Half of the CONTROL group (132 patients, 49.6%) complied with the advice of 2 liters intake within the 2 hours after the lumbar puncture. Of the FREE-FLUID group, 76 (27.9%) patients drank more than 0.5 liters (S3 Table).

### Occurrence of post-dural puncture headache

The rate of post-dural puncture headache within the 5 days after lumbar puncture was 33.1% in the FREE-FLUID group versus 38.0% in the CONTROL group with adjusted difference of ‒3.7% [95 CI, ‒11.6%; + 4.2%] (Table 2). The lower bound of confidence interval of the difference of post-dural puncture headache rate was below the non-inferiority margin of 10%, showing the noninferiority of the FREE-FLUID group compared to the CONTROL group (Fig 3).

**Table 2:** Occurrence of post-dural puncture headache.

	FREE-FLUID N = 276	CONTROL N = 278	Difference [95% CI] FREE-FLUID – CONTROL
Observed percentage (n)	**33.1% (89)**	**38.0% (100)**	
Adjusted percentage *	32.2%	36.0%	‒3.7% [‒11.6; + 4.2]
Adjusted percentage **	34.8%	41.4%	‒6.6 [‒14.7; + 1.5]
Adjusted percentage ***	34.8%	36.0%	‒1.2 [‒9.2; + 6.8]
Sensitivity analysis			
Adjusted percentage #1	33.1%	38.4%	‒5.4% [‒13.6; + 2.9]
Adjusted percentage #2	32.9%	38.3%	‒5.4% [‒13.7; + 2.8]
Adjusted percentage #3	30.5%	34.7%	‒4.2% [‒12.0; + 3.6]
*Missing*	*7*	*15*	

**Legend**:

*: Generalized linear model with center as random factor and missing data imputed as absence of post-dural puncture headache.

**: all missing data considered as PDPH events.

***: missing data considered as occurrence of PDPH in the FREE FLUID group and absence in the CONTROL group (worst case scenario)

#1: PP population

#2: ITT population and imputation multiple for missing data

#3: ITT population, missing data imputed as absence of post-dural puncture headache and ajustment on risk factors.

**Fig 3:**
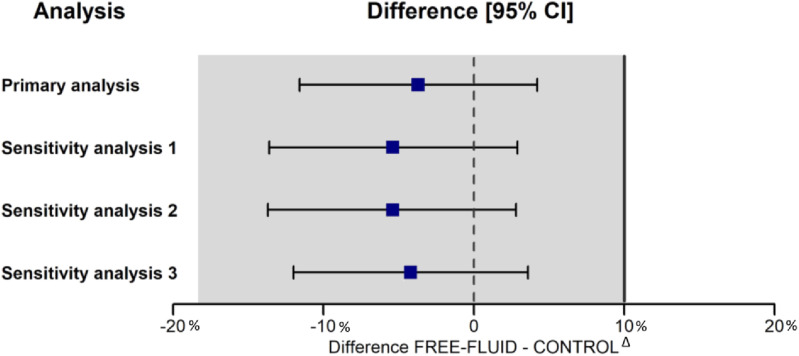
Difference of Post-dural Puncture Headache between FREE-FLUID group and CONTROL group. Legend: Sensibility analysis 3 on ITT population ITT and risk factors adjustment ∆ indicates the non-inferiority margin of 10% Primary analysis with ITT population and missing data imputed as absence of Post-dural Puncture Headache Sensibility analysis 1 on PP population Sensibility analysis 2 on ITT population and multiple imputation on missing data.

### Time-to-post-dural puncture headache onset

The mean time-to-post-dural puncture headache onset was 1.2 (SD 1.1) in the FREE-FLUID group, versus 1.1 (SD 0.9) in the CONTROL group (Table 3). The mean difference of 0.06 [95% CI: ‒0.23; + 0.35]; p = 0.7. The post-dural puncture headache onset was the day after the lumbar puncture for 49.4% of patients in the FREE-FLUID group versus 54.0% to the CONTROL group (S4 Figure).

**Table 3.** Characteristics of the Post-dural Puncture Headache in patients who experienced it.

Characteristics	FREE-FLUID N = 89	CONTROL N = 100	Difference* [95% CI] FREE-FLUID – CONTROL	p-value
Time-to-PDPH onset from LP (days)				
Mean (SD)	1.2 (1.1)	1.1 (0.9)	0.06 [‒0.23; 0.35]	0.68
Median	1	1		
Duration in days of the PDPH				
Mean (SD)	2.9 (1.6)	3.0 (1.6)	‒0.08 [‒0.53; 0.37]	0.72
Median	3	3		

**Legend**:

*:* * Linear mixed model with center as random effect. PDPH: post-dural puncture headache, LP: lumbar puncture, SD: standard deviation

The mean duration of post-dural puncture headache was 3.0 ± 1.6 in FREE-FLUID group versus 2.9 (SD 1.6) in CONTROL group. The mean difference of 0.06 [95% CI: ‒0.53; + 0.37]; p = 0.7 (Table 3).

Nine out of ten patients experienced post-dural puncture headache within two days of lumbar puncture. Post-dural puncture headache lasted 3 days in median in both groups: 3 [1.0; 4.0] days in the FREE-FLUID group versus 3 [1.5; 4.0] in the CONTROL group, difference ‒0.05; 95 CI [‒0.50; + 0.41] in favor of FREE-FLUID, p =  0.8).

At D5, 49 patients still had signs of post-dural puncture headache, resolving by D8. Of note, 24 patients still had persistent headache beyond D8 (S5 Table).

## Discussion

Our study is the first prospective multicenter randomized controlled trial comparing no advice of supplementation oral fluid intake, and recommendation of 2 liters of water intake within 2 hours after lumbar puncture to prevent a post-dural puncture headache. The confidence interval was below the non-inferiority margin of 10%, showing the non-inferiority of the FREE-FLUID group compared to the CONTROL group. The three sensitivity analyses were concordant (Fig 3). These findings are in accordance with Dieterich, who showed in 1988 in a superiority trial that hyperhydration with 2 liters of water was not efficient to prevent a post-dural puncture headache [15] . The strength of our study lies in the fact that we included 554 patients without modifying the organization of care or the hospital discharge schedule as recommended by Arevalo-Rodriguez [5].

Our research was based on a pragmatic trial design [18] so that the CONTROL group received care usually provided by nurses: encouraging patients to increase their intakes, without drastically controlling them. This design seems to be both more faithful to the practices carried out, and at the same time to correspond to the vision of personalized nursing care which take into account patient preference [19].

### Study Limitations

Our study presented some limitations. First, results were adjusted only for known risk factors of post-dural puncture headache [6]. It might have been interesting to adjust for indications of lumbar puncture, since patients scheduled for a lumbar puncture may have had time to find out about the examination. However, we take into account the “center” factor insofar as each center included only one type of patient: scheduled or unscheduled lumbar puncture. Thus, the use of a robust analysis model has made it possible to limit this pitfall. Second, in the absence of a previous study including a placebo group made it difficult to justify on clinical data the non-inferiority margin as recommended [16,17]. It was based on data from an exploratory and descriptive study carried out in our institution.

Finally, we defined the principal outcome by the 3^rd^ International Classification of Headache Disorders [1]. However, this definition lacks clarity on how to consider persistent headaches after 2 weeks [6]. In our study, only 24 patients presented headaches in the last call after D8.

### Implications for policy and practice

The results could be used to review the nursing advice given after a lumbar puncture. Although a supplementation of oral fluid intakes is not included in the latest recommendations for healthcare professionals in the field of post-dural puncture headache [6], several popular medical websites advise patients to increase their fluid intake after a lumbar puncture [20]. In our study, we observed that 76 patients in the FLUID-FREE group, (i.e., 27.9% of this group), drank more than the 500 ml bottle of water provided. These data may reflect patients’ belief in the benefits of preventive oral fluid supplementation in the post-dural puncture headache. However, it is known that the discrepancy between the advice read on the websites of large institutions, and the actual practices of carers can have a negative impact on patient confidence [21]. These results will enable us to update our knowledge of the prevention of post-dural puncture headache by supplementing the conclusions of the meta-analysis by Arevalo-Rodriguez et al. [5].

The evidence-based model is widely known, but nursing research often focuses more on innovation than on revising current practices [21]. However, there is a great proportion of non-evidence-based practice that nursing research could assess to determine its relevance [22]. In the field of care for people requiring lumbar puncture, stopping giving advice that is not useful and that limits patient comfort would free up time to better support the emotions associated with lumbar puncture which is still experienced as a pain-related trauma [23–25].

## Conclusion

Our study succeeded in showing the non-inferiority of the absence of specific advice on water intake after a lumbar puncture compared with advice to increase oral fluid to prevent a post-dural puncture headache.

This research could encourage nurses to identify non-evidence-based nursing practices in order to review them, and free up time to support relational, and comfort care.

## Supporting information

S1 Table Participating centers. (DOCX)

S2 Table Post-dural puncture headache advice examples provided to investigators for both groups of the study. (DOCX)

S3 Table Oral intakes within 2 hours after LP. (DOCX)

S4 Figure Day of PDPH onset according to the hydration group, no specific recommendation (FREE-FLUID) versus recommendation of hyperhydration (CONTROL). (DOCX)

S5 Table Prevalence of PDPH * within the 5 first days after DP. (DOCX)
