# Supplementary material for: Oral fluid supplementation for the prevention of post-dural puncture headache: A noninferiority randomized controlled trial
Source: PLoS One. 2025 Mar 12;20(3):e0319481. doi: 10.1371/journal.pone.0319481 (PMC11903041; doi:10.1371/journal.pone.0319481)
Supplement: S1 Table — (DOCX) [file pone.0319481.s001.docx]

**S1 Table - Participating centers**

|  | **FREE-FLUID N=276** | **CONTROL**  **N=278** | **Total N=554** |
| --- | --- | --- | --- |
| 1 – Nantes | 96 (34.8%) | 96 (34.5%) | 192 (34.7%) |
| 2 – La Roche sur Yon | 65 (23.6%) | 65 (23.4%) | 130 (23.5%) |
| 3 – Quimper | 48 (17.4%) | 48 (17.3%) | 96 (17.3%) |
| 4 – Le Mans | 41 (14.9%) | 40 (14.4%) | 81 (14.6%) |
| 5 – Cholet | 17 (6.2%) | 17 (6.1%) | 34 (6.1%) |
| 6 – Angers | 8 (2.9%) | 9 (3.2%) | 17 (3.1%) |
| 7 – Orléans | 1 (0.4%) | 3 (1.1%) | 4 (0.7%) |
| 8 – Saint-Nazaire | 0 (0.0%) | 0 (0.0%) | 0 (0.0%) |
