# Supplementary material for: Oral fluid supplementation for the prevention of post-dural puncture headache: A noninferiority randomized controlled trial
Source: PLoS One. 2025 Mar 12;20(3):e0319481. doi: 10.1371/journal.pone.0319481 (PMC11903041; doi:10.1371/journal.pone.0319481)
Supplement: S3 Table — (DOCX) [file pone.0319481.s003.docx]

**S3 Table – Oral intakes within 2 hours after LP**

| **Volume of intakes** | **FREE-FLUID group N= 276** |
| --- | --- |
| ≤500ml | 196 (72.0%) |
| >500ml | 76 (27.9%) |
| *Missing data* | *n =1* |
|  | **CONTROL group N= 278** |
| < 2000ml | 134 (50,1%) |
| = 2000ml | 132 (49,6%) |
| *Missing data* | *n=12* |
