# Supplementary material for: Oral fluid supplementation for the prevention of post-dural puncture headache: A noninferiority randomized controlled trial
Source: PLoS One. 2025 Mar 12;20(3):e0319481. doi: 10.1371/journal.pone.0319481 (PMC11903041; doi:10.1371/journal.pone.0319481)
Supplement: S4 Figure — (DOCX) [file pone.0319481.s004.docx]

**S4 Figure - Day of PDPH onset according to the hydration group, no specific recommendation (FREE-FLUID) versus recommendation of hyperhydration (CONTROL)**

**Legend:** PDPH: Post-dural Puncture Headache
