# Supplementary material for: Oral fluid supplementation for the prevention of post-dural puncture headache: A noninferiority randomized controlled trial
Source: PLoS One. 2025 Mar 12;20(3):e0319481. doi: 10.1371/journal.pone.0319481 (PMC11903041; doi:10.1371/journal.pone.0319481)
Supplement: S5 Table — (DOCX) [file pone.0319481.s005.docx]

**S5 Table - Prevalence of PDPH* within the 5 first days after DP**

|  | **FREE-FLUID N=89** | **FLUID-RECO**  **N=100** | **Total N=189** |
| --- | --- | --- | --- |
| D0 | 23 (25.8%) | 23 (23.0%) | 46 (24.3%) |
| D1 | 59 (66.3%) | 61 (61.0%) | 120 (63.5%) |
| D2 | 61 (69.3%) | 70 (70.0%) | 131 (69.7%) |
| D3 | 53 (60.2%) | 64 (64.0%) | 117 (62.2%) |
| D4 | 38 (43.2%) | 47 (47.0%) | 85 (45.2%) |
| D5 | 22 (24.7%) | 27 (27.0%) | 49 (25.9%) |

**Legend:** PDPH: Post-dural Puncture Headache

*: only patients with PDPH complying with the 2013 definition of PDPH
